# Supplementary material for: Groundwater Quality, Health Risk Assessment, and Source Distribution of Heavy Metals Contamination around Chromite Mines: Application of GIS, Sustainable Groundwater Management, Geostatistics, PCAMLR, and PMF Receptor Model
Source: Int J Environ Res Public Health. 2023 Jan 24;20(3):2113. doi: 10.3390/ijerph20032113 (PMC9916341; doi:10.3390/ijerph20032113)
Supplement: Supplementary file 1 [file ijerph-20-02113-s001.zip › ijerph-2102065-supplementary.pdf]

Supporting Information's

Groundwater Quality, Health Risk Assessment, and Source Distribution of Heavy Metals Contamination around Chromite Mines: Application of GIS, Sustainable Groundwater Management, Geostatistics, PCAMLR, and PMF Receptor Model

Abdur Rashid [1,5,*], Muhammad Ayub [2], Zahid Ullah [1], Asmat Ali [1], Tariq Sardar [3], Javed Iqbal [4], Xubo Gao [1,*], Jochen Bundschuh [4], Chengcheng Li [1], Seema Anjum Khattak [5], Liaqat Ali [5], Hamed A. El-Serehy [6], Prashant Kaushik [7] and Sardar Khan [8]

[1] State Key Laboratory of Biogeology and Environmental Geology, School of Environmental Studies,
China University of Geosciences, Wuhan, 430074, P.R. China
[2] Department of Botany Hazara University PO 21300 Pakistan
[3] Department of Environmental Sciences, Kohat University of Science and Technology, Pakistan
[4] School of Civil Engineering and Surveying, Faculty of Health, Engineering and Sciences, University of Southern Queensland, West Street, Toowoomba, 4350, Queensland, Australia
[5] National Centre of Excellence in Geology, University of Peshawar, 25130, Pakistan
[6] Department of Zoology, College of Science, King Saud University, Riyadh l1451, Saudi Arabia
[7] Instituto de Conservación y Mejora de la Agrodiversidad Valenciana, Universitat Politècnica de València, 46022 Valencia, Spain
[8] Department of Environmental Sciences, University of Peshawar, PO 25120, Pakistan
* Correspondence: abdur.rashid@bs.qau.edu.pk (A.R.), Xubogao@qq.com (X.G.)

Table S1. Compile principal component analysis (PCA) results of groundwater (n=55), in mining, non-mining region, and chromite mines water collected from Malakand, Northern Pakistan.

**Parameters**	**Groundwater (n=55)**					**Chromite mines water (n=5)**		
Factors	F1	F2	F3	F4	F5	F1	F2	F3
pH	**0.949**	0.103	-0.097	-0.148	0.093	**0.531**	**-0.728**	0.421
Depth	0.011	-0.285	-0.302	**0.650**	-0.059	**-0.700**	0.097	**0.673**
Elevation	-0.149	0.070	**-0.498**	0.287	0.420	**0.551**	0.463	-0.003
EC	**0.604**	**0.849**	-0.101	0.052	**-0.561**	**0.682**	-0.188	**0.707**
Temperature	-0.335	-0.197	**-0.521**	**-0.600**	-0.025	**0.874**	0.402	-0.236
TDS	**0.559**	**0.853**	-0.134	0.169	**-0.512**	0.356	-0.220	**0.903**
Turbidity	-0.200	0.256	-0.318	0.055	**0.673**	**-0.934**	0.060	-0.311
Na	**0.985**	0.024	0.015	0.043	0.072	-0.029	**0.917**	0.396
K	**0.686**	-0.187	0.195	-0.355	0.230	**0.966**	-0.049	0.255
Mg	**-0.858**	-0.244	0.171	-0.055	0.054	-0.145	**-0.758**	**-0.633**
Ca	**-0.909**	0.141	0.206	0.121	-0.004	0.324	**-0.653**	-0.447
PO_4	-0.020	0.030	-0.026	**-0.620**	-0.286	**0.819**	-0.167	-0.116
NO_3	0.206	-0.107	**-0.680**	0.232	0.000	**-0.503**	**-0.805**	0.252
HCO_3	**0.900**	0.197	0.110	-0.058	0.237	**-0.520**	**0.639**	**-0.566**
Cl	0.065	0.038	**0.749**	0.254	0.034	0.312	**0.728**	**0.553**
SO_4	**0.501**	-0.215	**0.762**	0.322	0.214	0.104	**0.567**	-0.403
ORP	-0.140	**0.659**	-0.105	-0.071	**0.622**	**0.939**	-0.016	-0.244
Cr	**0.543**	-0.275	-0.375	**0.514**	-0.074	**0.973**	-0.186	0.134
Ni	**-0.534**	0.350	0.197	-0.308	**0.536**	**-0.514**	**-0.769**	0.038
Mn	**0.527**	**0.594**	0.211	0.275	-0.005	**0.965**	0.259	-0.045
Eigenvalue	5.288	2.848	2.713	2.062	1.752	8.437	5.845	3.867
Variability (%)	26.439	14.239	13.563	10.311	8.759	42.184	29.224	19.333
Cumulative %	26.439	40.678	54.240	64.551	73.310	42.184	71.408	90.741

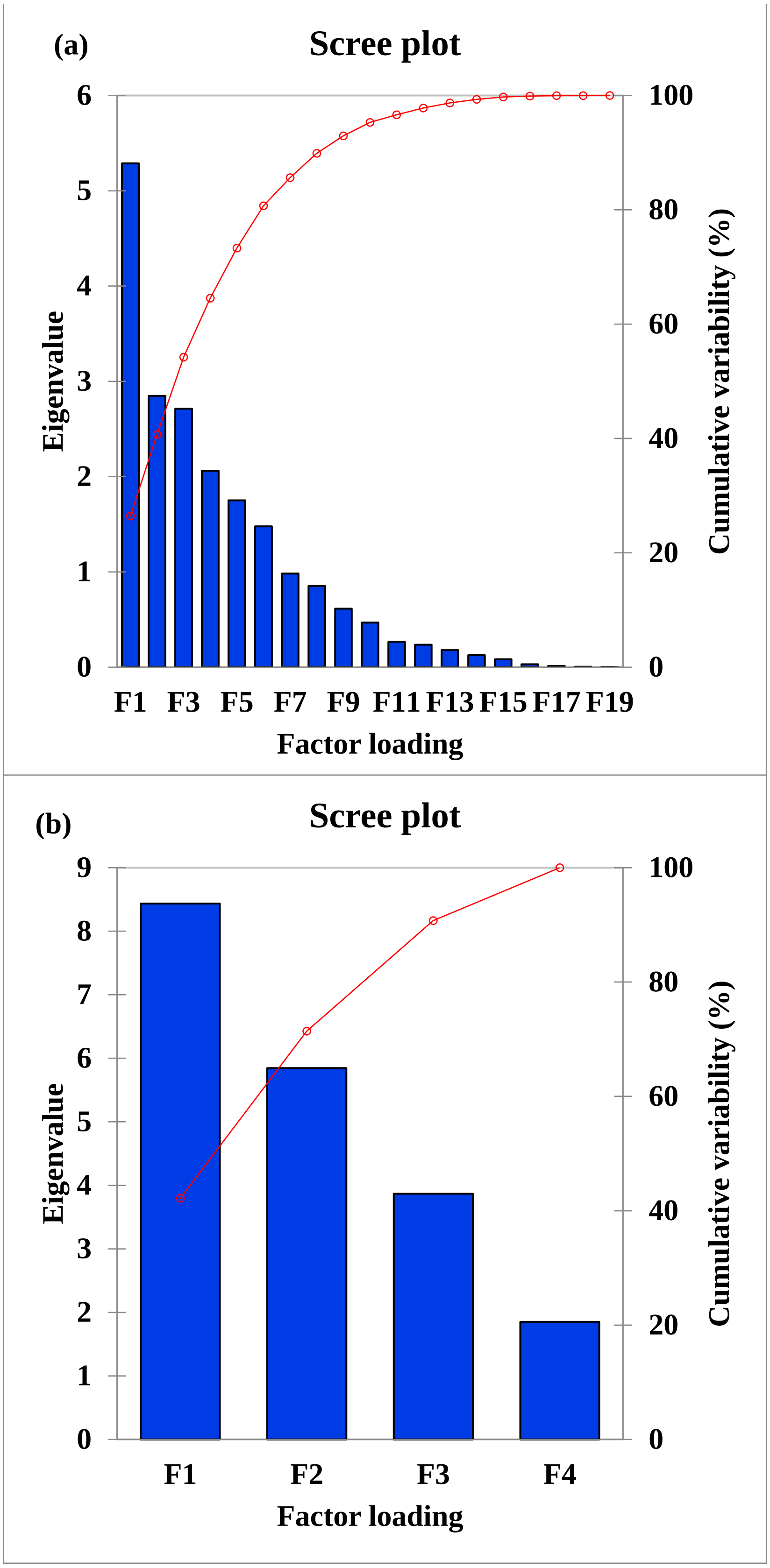

Figure S1. (a) Overall loading factors of PCA in groundwater of mining, and non-mining region, and (b) loading factors of PCA in chromite mines water in ultramafic terrain of Malakand, Pakistan.

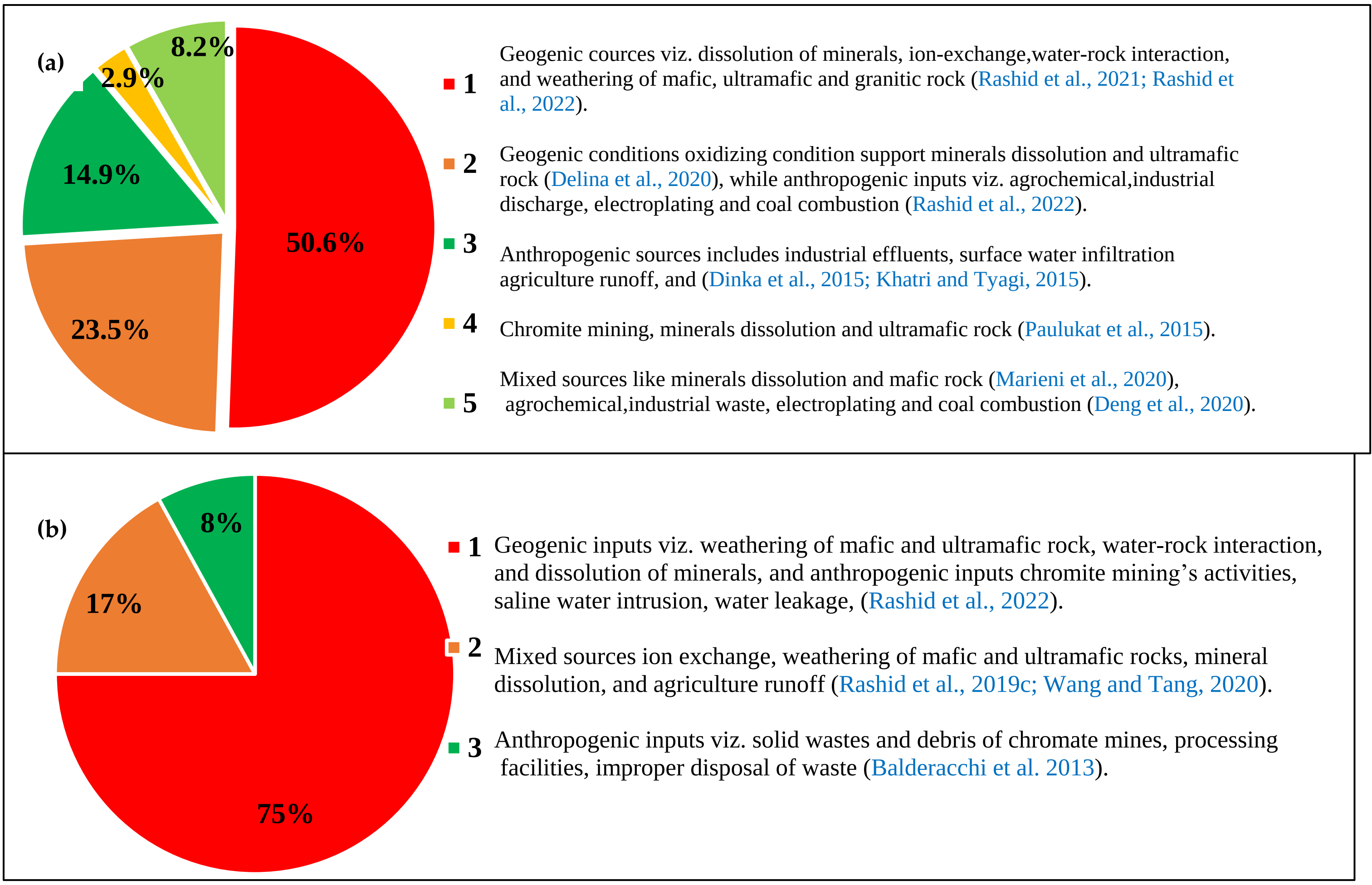

Figure S2. (**a**) Showing PCA-MLR results of groundwater sources, and (**b**) chromite mines water percentage contribution of pollutant sources in the groundwater of the study are
